# An Integrated Framework for Drought Stress in Plants

**DOI:** 10.3390/ijms25179347

**Published:** 2024-08-28

**Authors:** Yanyong Cao, Wenbo Yang, Juan Ma, Zeqiang Cheng, Xuan Zhang, Xueman Liu, Xiaolin Wu, Jinghua Zhang

**Affiliations:** 1 Institute of Cereal Crops, Henan Academy of Agricultural Sciences, The Shennong Laboratory, Zhengzhou 450002, China; bbg.123@163.com (W.Y.); majuanjuan85@126.com (J.M.); zeqiangcheng@163.com (Z.C.); 2 National Key Laboratory of Wheat and Maize Crop Science, College of Life Sciences, Henan Agricultural University, Zhengzhou 450002, China; zhangxuan0280@163.com (X.Z.); 13783990426@163.com (X.L.); wuxiaolin@henau.edu.cn (X.W.)

**Keywords:** plants, drought, osmotic regulation, transcription factor, reactive oxygen species (ROS), phytohormone, sRNA

## Abstract

With global warming, drought stress is becoming increasingly severe, causing serious impacts on crop yield and quality. In order to survive under adverse conditions such as drought stress, plants have evolved a certain mechanism to cope. The tolerance to drought stress is mainly improved through the synergistic effect of regulatory pathways, such as transcription factors, phytohormone, stomatal movement, osmotic substances, sRNA, and antioxidant systems. This study summarizes the research progress on plant drought resistance, in order to provide a reference for improving plant drought resistance and cultivating drought-resistant varieties through genetic engineering technology.

## 1. Introduction

Wheat (*Triticum aestivum* L.), corn (*Zea mays* L.), and rice (*Oryza sativa* L.) are the most important food crops, as well as crucial feed and energy crops, playing a significant role in the development of the economy [1]. However, in nature, due to differences in climate and geographical location, economic crops are not always living in suitable environments. Once the habitat exceeds the range in which plants can maintain normal growth, it will cause varying degrees of damage to the plants. We refer to these environmental factors that can alter the conditions for normal growth and development as adversity, also known as stress [2]. These abiotic stresses not only limit the geographical distribution of plants but also reduce the yield and quality of important crops [2].

Due to sessile growth, most plants cannot avoid unfavorable environments by moving as animals do. Therefore, over the long course of evolution, plants have developed highly complex mechanisms to respond to and resist adverse conditions [3]. In the face of abiotic stress, plants must continuously sense environmental changes, respond promptly, and undergo corresponding physiological and biochemical changes to cope with the adverse conditions [3]. Among the various abiotic stresses, drought has a particularly significant negative impact on crops, posing a major threat to sustainable crop production. It is a primary factor leading to reduced yields of food crops and a decline in agricultural productivity [2]. For important grain and cash crops, drought conditions can alter flowering periods, cause abnormal growth, and ultimately result in reduced yields or even total crop failure, posing a serious threat to food security [3].

Prolonged stress can lead to metabolic abnormalities in plants. In plants, physiological and metabolic mechanisms have developed to help alleviate drought stress. These metabolic systems can activate gene regulatory networks, enhancing the adaptability and resistance to environmental challenges [4]. Therefore, studying abiotic stress and the mechanisms of plant responses to various stresses is a crucial topic in botanical research. This review summarizes the research progress on plant drought resistance, in order to provide a reference for improving plant drought resistance and cultivating drought-resistant varieties through genetic engineering technology.

## 2. Damage to Plants from Drought

**Figure 1:**
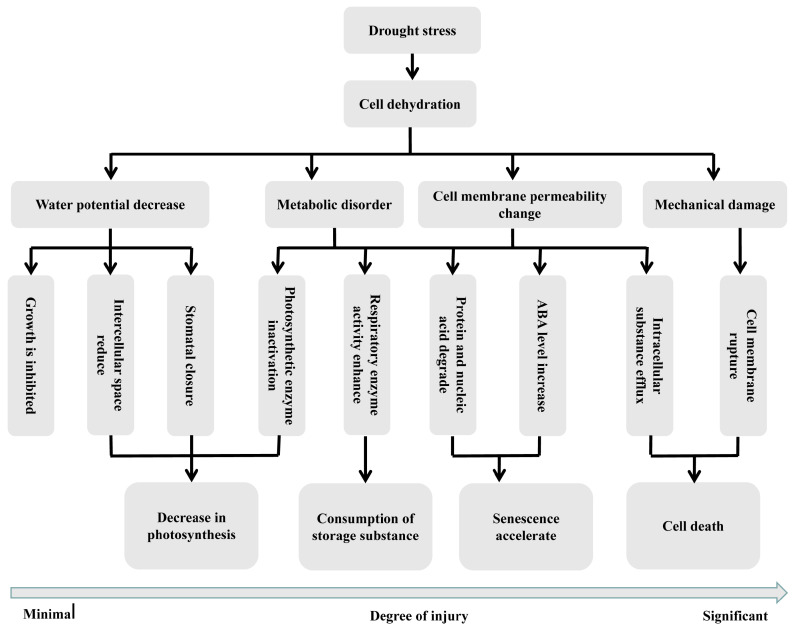
Drought injury to plant cells. Changes caused by drought stress to plants, including water potential decrease, metabolic disorder, cell membrane permeability change and mechanical damage.

Water is a crucial medium within cells and a fundamental substance for cell division and growth in plants. Plant growth and development are highly sensitive to water deficiency. The essence of the damage caused by drought stress to plants is protoplasm dehydration. Under drought stress, due to insufficient available water, the water lost through transpiration is not restocked, leading to the disorganization of the bilayer arrangement of membrane lipid molecules. The plasma membrane contracts, forming gaps and cracks, which alter cell permeability, cause leakage of cytoplasmic solutes, and inhibit plant growth. The increased concentration of cellular electrolytes due to protoplasm dehydration results in metabolic disorders within the cells (Figure 1) [5].

Photosynthesis is the first physiological process affected by drought stress. Under drought conditions, the stomata of leaves close, reducing CO_2_ absorption. The activity of Calvin-cycle enzymes decreases, the ratio of photorespiration to dark respiration increases, and the photosynthetic apparatus is damaged, ultimately leading to a significant decline in photosynthetic efficiency [6].

Prolonged drought stress leads to the excessive accumulation of free radicals within plants. Under normal conditions, their production and removal are in a dynamic balance. However, under drought stress, the defense and scavenging systems cannot promptly eliminate free radicals, resulting in the lipid peroxidation of cell membranes. Malondialdehyde (MDA) is the final product of lipid peroxidation, which disrupts the stability of cell membranes, reduces the content of unsaturated fatty acids in the membrane, and decreases membrane fluidity [7,8].

To survive under adverse conditions such as drought stress, plants have evolved numerous mechanisms to cope with unfavorable environments. Plants primarily enhance their tolerance to drought stress through the coordinated action of transcription factors, phytohormone, stomatal movement, osmotic substances, sRNA, and antioxidant systems [2,9].

## 3. Osmotic Regulation under Drought Stress

The direct damage caused by drought stress to plants is osmotic stress, which leads to an imbalance in cell turgidity and restricts plant growth [3]. In response to the adverse effects of drought stress, plants tend to synthesize and accumulate osmotic regulatory substances, such as mannitol, sorbitol, soluble sugars, polyamines, free amino acids, trehalose (Tre), betaine, and proline. These substances help reduce the osmotic potential of cells, thereby minimizing water loss and protecting the stability of cell membranes and proteins (Table 1) [10].

### 3.1. Soluble Proteins

Drought stress can induce changes in both the quality and quantity of soluble proteins in plants. Heat shock proteins (HSPs), osmotic proteins, and late embryonic abundant proteins (LEAs) are soluble proteins that are significantly produced and accumulated during the defense against drought stress [11]. LEA proteins, classified as “hydrophilic proteins”, protect other proteins from the effects of osmotic stress [12]. HSPs aid in binding, folding, displacing, and degrading other proteins [13]. Osmotic regulatory proteins protect cells from metabolic disorders and the effects of osmotic stress [14].

Dehydrins or type II LEA proteins play a crucial role in osmotic regulation. During drought conditions, these proteins accumulate along with other LEA proteins, contributing to the regulation of membrane protein stability and osmotic potential [15]. Dehydrins not only protect cells from dehydration stress similar to glycine betaine (GB), proline, and sucrose but also participate in ion chelation and solute concentration regulation in the cytoplasm under drought conditions [16,17]. They can also maintain the stability of macromolecules by binding water molecules to their hydrophilic surfaces, thereby inhibiting or reversing further protein denaturation [18]. Studies have shown that drought-resistant varieties of different crops significantly increase the accumulation of dehydrins in root and leaf tissues under drought conditions, thus protecting them from further dehydration damage [19].

Osmotin is a PR-5 protein rich in cysteine that can reduce plant water potential under osmotic stress, protecting plant membranes from damage [20]. Experimental evidence showed that the overexpression of osmotin genes from tobacco in mulberry (*Morus alba* L.) enhanced cell membrane stability and photosynthetic yield [21]. Similarly, transgenic tomato plants overexpressing the osmotin gene exhibited increased tolerance to drought stress [22].

Plants produce HSPs ranging in size from 15 to 104 kDa in response to temperature increases of 5–10 °C. In early studies, HSPs were described as proteins whose concentrations significantly increase when plants grow at higher temperatures. However, recent research indicated that HSPs aided in folding newly synthesized proteins or preventing protein misfolding [23]. Studies have found that the small heat shock protein (sHSP) CaHsp25.9 enhanced the tolerance to drought stress by reducing ROS levels, enhancing antioxidant enzyme activity and increasing the expression of stress-related genes in pepper [24].

### 3.2. Soluble Sugars and Sugar Alcohols

Carbohydrates participate in regulating plant cellular energy metabolism and growth and development. Drought stress increases the content of reducing sugars, such as glucose, fructose, sucrose, and raffinose, as well as sugar alcohols like mannitol and sorbitol [25]. Soluble sugars (SSs) play roles in membrane protection and detoxifying harmful ROS. Rapid accumulation of SS and sugar alcohols under drought stress can significantly reduce cell osmotic potential and enhance plant water retention capacity [26].

Sucrose is an important substance regulating stress response and tolerance mechanisms, serving as a primary carbohydrate transported from source organs to sink organs in plants and converted into other products. It is a crucial component in regulating plant drought resistance [27]. Experimental evidence demonstrated that drought-tolerant seedlings increased sucrose synthesis at metabolic and gene expression levels to enhance energy storage. Therefore, sucrose accumulation is a key characteristic for drought tolerance in crops like maize [28]. Tre is a non-reducing disaccharide of glucose that can protect plants from various stress injuries. It safeguards cellular components, acts as a signaling molecule and antioxidant, and serves as an inducer of stress response genes [29,30]. Studies confirmed that exogenous Tre could effectively maintain osmotic balance in plants under drought stress [31]. Transgenic plants with genes for Tre biosynthesis also exhibited enhanced tolerance to drought stress [32].

Sugar alcohols mitigate the adverse effects of plant growth under drought stress by regulating osmotic balance to maintain the cellular water content. They also function at the cellular level by sequestering Na^+^ into vacuoles or extracellular spaces [33]. Sugar alcohols provide protection to cell structures through interactions with various enzymes and membrane protein complexes [34]. Experimental studies demonstrated that mannitol and sorbitol participate in osmotic regulation, scavenging stress-induced oxygen free radicals, reducing plant osmotic potential, minimizing water loss during drought stress, and enhancing plant drought tolerance [35].

### 3.3. Glycine Betaine

Studies show that the accumulation of glycine betaine (GB) is beneficial for plants to adapt to drought stress. Under water stress conditions, various economically important crops such as rice, maize, and sunflower produce GB [36]. GB functions to protect membranes and enzymes, thereby maintaining the stability of the Photosystem II (PS II) complex under adverse environmental conditions [37].

In higher plants, *choline monooxygenase* (*CMO*) and *betaine aldehyde dehydrogenase* (*BADH*) are key genes involved in GB biosynthesis. Cis-regulatory elements in the regions upstream of these genes participate in transcriptional regulation of various biological processes in response to drought stress [38]. For instance, cis-regulatory elements responsive to stress and hormones have been identified in the synthesis genes of GB in watermelon [39]. BADH and choline oxidase (COD) are crucial enzymes mediating GB synthesis in plants that do not naturally accumulate GB. The introduction of these genes of enzymes can confer GB-like functional traits to non-GB accumulating crops [40].

### 3.4. Polyamines

Polyamines (PAs) are a class of low-molecular-weight biogenic amines that regulate cellular pH and are widely present in plants. They play roles in signal transduction and gene expression regulation in response to various stresses. Plants increase the PA levels in response to different stresses [41]. For example, in rapeseed leaves, PA levels significantly increase under osmotic stress, and the accumulation occurs before proline accumulation [42]. The involvement of PAs in these processes may relate to pathways that require glutamate as a precursor for both proline and PA biosynthesis, leading to significant changes in the PA pool [42].

### 3.5. Proline

Proline is an amino acid synthesized by plants under stress conditions, playing a crucial role in osmotic regulation. The biosynthesis of proline requires the involvement of glutamate kinase (GK), and the activity of this enzyme significantly increases under drought conditions [43]. Research has shown that sunflower plants grown under water stress exhibited significantly higher GK activity compared to plants grown under normal conditions [44].

Proline plays significant roles in higher plants: it reduces cellular water potential, enhances leaf water content, stabilizes membranes and protein conformations, and reduces photooxidative damage to thylakoid membranes through ROS scavenging [45]. Under drought conditions, the proline content in many plants can increase over 100-times compared to normal levels, constituting up to 80% of the total amino acid pool [46]. Studies have found that as the severity of stress increased, the levels of proline and other osmotic regulators also increased, helping maize maintain normal relative water content [47]. There is a high correlation between plant stress resistance and proline accumulation; even within the same species, more tolerant varieties tend to have higher proline levels compared to sensitive ones. The accumulation of proline has been correlated with drought tolerance in both maize and barley under drought stress [48]. Plants grown under water-deficit conditions show higher proline levels, significantly lower lipid peroxidation, and markedly increased antioxidant enzyme activity [49]. These findings suggest that proline not only functions as an osmotic regulator but also serves as a precursor for free radical scavenging. Therefore, conducting new metabolic studies around this theme holds special significance for enhancing crop drought tolerance.

**Table 1:** Osmolites involved in drought stress.

Osmolites	Chemical Composition	Role	Reference
Soluble proteins	LEAs	Protecting other proteins from the effects of osmotic stress	[12]
	HSPs	Aiding in binding, folding, displacing, and degrading other proteins	[13]
	Osmotic proteins	Protecting cells from metabolic disorders and the effects of osmotic stress	[14]
Soluble sugars	Carbohydrate	Playing roles in membrane protection and detoxifying harmful ROS	[25]
Sugar alcohols	Mannitol and sorbitol	Reducing cell osmotic potential and enhance plant water retention capacity	[26]
Glycine betaine	Alkaloid	Protecting membranes and enzymes	[37]
Polyamines	Biogenic amines	Play roles in signal transduction and gene expression regulation in response to various stresses	[41]
Proline	Amino acid	Playing roles in osmotic regulation	[45]

## 4. Regulation Involving Transcription Factors

**Figure 2:**
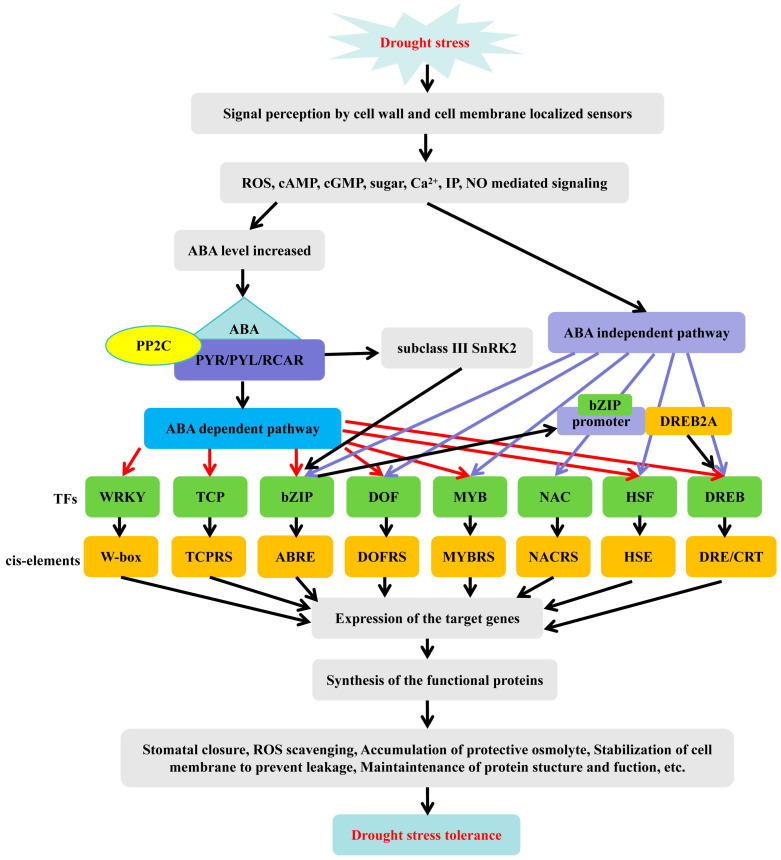
Schematic diagram of transcription factors sensing drought stress signals. ABA: abscisic acid; Ca^2+^: calcium ion; cAMP: cyclic adenosine monophosphate; cGMP: cyclic guanosine monophosphate; IP: phosphoinositide; NO: nitric oxide; PP2C: protein phosphatase; ROS: reactive oxygen species; SnRK2: SNF1-related protein kinase involved in ABA signaling. Red arrows represent ABA dependent pathway; Blue arrows represent ABA dependent pathway.

Transcription factors (TFs) are regulatory factors in plants that respond to stress by interacting with the promoters of target genes to regulate their expression. TFs mediate responses to abiotic stresses by regulating a series of intracellular signaling processes. When facing drought stress, plant cells sense stimulus signals and activate stress-responsive genes to combat the stress [50]. Numerous studies have shown that TFs, such as NAC, WRKY, bZIP, HD-ZIP, DREB/CBF, and MYB, play crucial roles in enhancing plant drought resistance (Figure 2). They collaborate across various biological processes to ensure appropriate biochemical responses that aid plants in adapting to adverse environments.

TFs perceive environmental stress stimuli through sensors located on the plant cell wall and plasma membrane and then transduce extracellular signals into the cell through various second messengers within the cell, such as ROS, cyclic nucleotides (cAMP, cGMP), sugars, calcium ions (Ca^2+^), inositol phosphates, and nitric oxide (NO) [13,51]. Signal transduction mediated by these second messengers activates calcium-dependent protein kinases (CDPKs), mitogen-activated protein kinases (MAPKs), and various phosphatases, which, in turn, activate or inhibit the activity of target transcription factors through phosphorylation or dephosphorylation processes [52].

### 4.1. bZIP Transcription Factors

The basic leucine zipper (bZIP) family is a widely distributed class of TFs in plants. These TFs are characterized by a highly conserved bZIP DNA-binding domain, which contains two functional regions: a conserved N-terminal domain and a variable leucine zipper region at the C-terminus [53]. Genes belonging to the A subfamily of bZIP enhance plant tolerance to abiotic stress in an ABA-dependent manner. For instance, *ZmbZIP72* was induced by ABA and drought treatment, and overexpression of *ZmbZIP72* in transgenic Arabidopsis (*Arabidopsis thaliana*) enhanced drought tolerance [54]. Furthermore, the overexpression of *ZmbZIP72* increased the expression of ABA-inducible genes, such as *RD29B*, *RAB18*, and *HIS1-3* [54]. bZIP TFs also regulate drought stress responses through negative modulation of ABA signaling. For example, the tomato *SlbZIP38* gene was downregulated under drought stress and ABA treatment, and the overexpression of *SlbZIP38* in transgenic tomatoes increased sensitivity to drought and salt stress tolerance [55]. Similarly, pepper *CaATBZ1*, subgroup A bZIP TF, negatively regulated ABA signaling and drought stress response, since silencing *CaATBZ1* in pepper plants led to enhanced drought tolerance via ABA-mediated signaling [56]. These findings indicate that bZIP TFs play a crucial role in enhancing tolerance to abiotic stress through the modulation of ABA signaling pathways.

### 4.2. DREB Transcription Factors

The dehydration-responsive element (DRE) is part of the promoter sequences of many stress-responsive genes. Proteins that bind to DRE are named DREBs. DREBs have been identified in various plants and participate in abiotic stress responses in both ABA-dependent and ABA-independent manners. DREBs contain a highly conserved apetala2/ethylene response factor (AP2/ERF) DNA-binding domain. While DREBs typically recognize DRE sequences, some members can also recognize the AGCCGCC cis-element, known as the GCC box. Drought stress induces the upregulation of many transcription factor genes, including DREBs. For example, the maize AP2/ERF family member *ZmEREBP60* was highly induced by drought in roots, coleoptiles, and leaves. The overexpression of *ZmEREBP60* enhanced drought tolerance in transgenic plants, alleviating the drought-induced accumulation of H_2_O_2_ and increased MDA levels [57]. The A2-type DREB gene *ZmDREB2.9* showed significantly increased expression in response to drought and ABA treatment, playing a crucial role in maize stress responses [58].

Different DREB proteins are induced by various abiotic stresses. When DREB TFs are overexpressed in transgenic plants, they also induce the expression of certain stress-responsive genes, leading to overall fitness advantages of transgenic plants under diverse environmental stress conditions. For example, in Arabidopsis, overexpressing *AtDREB1A* led to the expression levels of 12 stress-responsive genes being significantly upregulated compared to wild-type plants [59]. Similarly, in transgenic Arabidopsis plants overexpressing *OsDREB1A*, the expression of six stress-related genes also increased [60]. Therefore, DREBs serve as key regulators in plants responding to abiotic stress, and the overexpression of *DREBs* can significantly enhance plant tolerance to abiotic stresses.

### 4.3. MYB Transcription Factors

The MYB protein superfamily is part of a large family of TFs found in nearly all eukaryotic organisms, performing various functions [61].

Research has confirmed that multiple MYB genes are upregulated under drought stress. For instance, *ZmMYB3R* was induced by both drought and ABA. *ZmMYB3R* is a positive regulatory TF that enhanced plant drought tolerance through an ABA-dependent pathway. Transgenic Arabidopsis overexpressing *ZmMYB3R* showed enhanced growth performance, higher survival rates, and increased enzyme activities of catalase (CAT), peroxidase (POD), and superoxide dismutase (SOD) [62]. *ZmMYB-CC10* enhanced maize drought tolerance by reducing oxidative damage. The overexpression of *ZmMYB-CC10* increased the expression of *ZmAPX4* under drought stress [63]. Luciferase assays indicated that ZmMYB-CC10 directly bound to the promoter of *ZmAPX4* to activate its expression, thereby enhancing APX activity and reducing H_2_O_2_ levels [63]. The overexpression of *OsMYB55* in maize reduced the adverse effects of high temperature and drought stress, resulting in higher plant biomass and less leaf damage compared to wild-type plants [64].

MYB transcription factors are involved in ABA-dependent drought stress responses [65]. For example, MYB2 in Arabidopsis interacted with the promoter region of the dehydration-responsive gene *rd22*, inducing the expression of multiple ABA-responsive genes [66]. The overexpression of *OsMYB48-1* in rice triggered the upregulation of numerous ABA biosynthesis, early signaling, and late responsive genes under drought stress, resulting in improving tolerance to drought [67]. In summary, MYB transcription factors cooperate with ABA signaling to regulate plant drought stress tolerance, acting as positive regulators of drought tolerance.

### 4.4. NAC Transcription Factors

NAC (NAM, ATAF1,2, and CUC2) transcription factors play crucial roles in regulating plant growth, development, and responses to drought stress [68]. NAC proteins enhance drought resistance by activating the expression of stress-responsive genes, promoting stomatal closure, and increasing osmolyte accumulation [69]. In maize, the overexpression of *ZmNAC111* significantly improved drought tolerance, and natural variation in the *ZmNAC111* promoter was closely associated with drought resistance [70]. *ZmNAC49* was rapidly induced by drought stress, and the overexpression of *ZmNAC49* in maize led to reduced stomatal conductance and density to enhance drought stress [71]. A further study revealed that ZmNAC49 directly bound to the promoter of *ZmMUTE*, a gene related to stomatal development, to suppress its expression, thereby enhancing drought resistance by decreasing stomatal density [71]. The overexpression of *ZmNST3*, a novel NAC transcriptional factor, also enhanced maize drought tolerance [72]. It was demonstrated that ZmNST3 affected the expression of genes related to the synthesis of antioxidant enzyme secondary metabolites that could enhance drought resistance [72].

In rice, transcriptomic studies demonstrated that *OsNAC1* was upregulated during drought stress and ABA treatment. The overexpression of *OsNAC1* protected against drought stress, resulting in increased fertile spikelets under drought conditions. *OsNAC1*-overexpressing plants maintained higher water retention capacity by mediating stomatal closure without affecting normal photosynthetic rates. Comparative transcriptome analysis revealed the upregulation of approximately 40 genes related to drought tolerance in *OsNAC1*-overexpressing plants compared with the wild type [73].

In Arabidopsis, the H_2_O_2_-induced NAC transcription factor JUNGBRUNNEN1 (AtJUB1) was involved in regulating ROS signaling and mediating drought stress responses [74]. *AtJUB1* directly triggered the expression of the AP2-type transcription factor *DREB2A* associated with drought stress response [74]. *AtJUB1* also acted as a negative regulator of GA or BR biosynthesis genes, leading to decreased levels of these plant hormones and stabilizing DELLA proteins [74]. Similarly, the homologous protein encoded by *SlJUB1* in tomato directly bound to the promoter of *SlDREB* and *DELLLA* genes to regulate ROS homeostasis and the expression of stress-related genes, thereby playing a crucial role in drought stress tolerance [75].

NAC transcription factors are localized in the nucleus. However, due to the presence of transmembrane domains, some NAC transcription factors are localized in the endoplasmic reticulum or membrane regions and are, therefore, referred to as NTLs (NAC-TF associated with transmembrane motif1-like). Studies have found that during stress responses, these NTLs are transported to the nucleus through various post-translational modifications, including phosphorylation, membrane protein cleavage, and alternative splicing [76]. In *Medicago falcata*, MfNACsa was membrane-bound under unstressed conditions, but during drought stress, MfNACsa was transported to the nucleus through de-S-palmitoylation [77], regulating the transcriptional module of GLYOXALASE1 to reduce cell osmotic potential during drought stress. In summary, these NAC transcription factors integrate multiple pathways and serve as candidate genes for enhancing crop drought tolerance.

### 4.5. WRKY Transcription Factors

WRKY transcription factors are involved in responses to various stresses [78,79]. They are induced by drought stress and are associated with phytohormone signals, MAPK kinase signaling pathways, and self-regulation of WRKYs [80].

WRKY genes act as activators of ABA signaling, positively regulating stomatal closure mediated by drought stress. These transcription factors integrate various stress signals downstream to modulate stress responses in plants. The overexpression of *ZmWRKY79* in Arabidopsis enhanced the survival rate under drought stress, accompanied by increased lateral roots, reduced stomatal aperture, and minimized water loss [81]. *ZmWRKY79* also promoted ROS scavenging, reducing the accumulation of H_2_O_2_ and MDA while increasing antioxidant enzyme activities [81]. *ZmWRKY40* was induced by drought and ABA, rapidly responding to drought stress. The overexpression of *ZmWRKY40* enhanced drought tolerance in transgenic Arabidopsis by regulating the expression of stress-related genes, alongside increasing activities of POD and CAT under drought stress to lower ROS levels [82]. *ZmWRKY104*, a substrate of *ZmMPK6*, enhanced drought tolerance when overexpressed in maize, alleviating oxidative damage induced by drought [83]. Notably, the overexpression of *OsWRKY11* driven by the promoter of the heat shock protein gene *OsHSP101* resulted in exhibiting both drought and heat tolerance phenotypes in transgenic lines [84]. These findings underscore the proactive role of WRKYs in responding to abiotic stresses, offering candidate genes for breeding drought tolerance in crops like maize.

### 4.6. Heat Shock Transcription Factors (HSFs)

Heat shock transcription factors (HSFs) are a class of transcription factors widely present in plants, primarily induced by abiotic stresses such as high temperature and drought [85]. In response to these stresses, HSFs regulate phytohormone and non-biological signaling pathways, playing crucial roles in the modulation of stress-responsive gene expression [86]. Studies have shown that *ZmHsf08* was induced by drought and ABA treatment [87]. The overexpression of *ZmHsf08* in maize increased sensitivity to salt and drought stresses, exhibiting reduced survival rates, elevated ROS levels, and increased MDA contents compared with WT, indicating that *ZmHsf08* negatively regulated drought stress [87]. Furthermore, research has demonstrated that transgenic plants overexpressing *ZmHsf06* showed enhanced drought stress tolerance as higher activities of SOD, POD, and CAT compared to WT [88].

During abiotic stress processes, ABA signaling crosstalks with HSF-mediated gene expression. For instance, the expression level of *AtHsfA6a* increased with enhanced external ABA and drought signals, and the overexpression of *AtHsfA6a* exhibited hypersensitivity to ABA and enhanced tolerance against drought stress [89]. In transgenic plants overexpressing *HsfA2*, dehydration-responsive genes such as *AtGolS* and *RafS* accumulated significantly [90]. The expression of *HsfA3* in response to drought and high-temperature stresses depended on *DREB2A* expression [91]. Concurrently, AtHsfA3 and AtHsfA1b participated in different signaling pathways to enhance plant tolerance to drought stress [90]. Therefore, HSFs are among the key transcription factors influencing the expression of multiple stress-responsive genes.

## 5. ROS

Drought stress causes cellular dehydration, leading to osmotic and oxidative stress. The perception of stress signals triggers a cascade of responses that ultimately results in the production of ROS. Superoxide anion (O_2_^•−^), hydrogen peroxide (H_2_O_2_), hydroxyl radicals (^•^OH), and singlet oxygen (^1^O_2_) are ROS formed in plants in response to the reduction of oxygen molecules. Excessive ROS can cause cellular damage and death [92,93,94].

During normal plant growth, the levels of ROS inside organelles are relatively low. However, when adversity strikes, disturbances in cell water potential and metabolism lead to an increase in ROS levels, disrupting cellular homeostasis [95]. The balance between ROS generation and scavenging mechanisms maintains ROS homeostasis, which plays a crucial role in protecting plants from stress-induced damage [96]. Therefore, understanding the mechanisms of ROS production, scavenging, and signal transduction can provide new insights into enhancing crop tolerance to adverse environmental conditions.

### 5.1. The Physiological Roles of ROS

The physiological roles of ROS vary in plants, serving both as signaling molecules involved in regulating growth and development processes and as part of defense mechanisms against biotic and abiotic stresses. In growth and development, moderate levels of ROS participate in regulating processes, such as seed germination, root development, flowering, and fruit ripening [97]. During stress responses, the rapid accumulation of ROS acts as an early signal triggering a cascade of defense reactions, including activation of antioxidant systems, scavenging of ROS, activation of signaling pathways, and modulation of gene expression [98]. However, excessive ROS levels can lead to cell damage and death, underscoring the importance of maintaining appropriate ROS levels for plant growth and survival.

In plant cells, chloroplasts, mitochondria, peroxisomes, plasma membranes, and cell walls are the main sites of ROS production [99,100]. ROS metabolism is crucial for the growth and adaptation of crops under stress conditions. The generation and removal of ROS are essential factors in plant defense processes.

Under normal conditions, ROS can serve as second messengers or signaling molecules, transmitting signals to the nucleus through redox reactions. ROS increase tolerance to various abiotic stresses through pathways like Mitogen-Activated Protein Kinases (MAPKs) and so on [101]. H_2_O_2_, a byproduct of ROS cleared by superoxide dismutase (SOD), modulates Ca^2+^ and NO signaling pathways, playing a role in responses to diverse abiotic stresses and serving as a crucial component in regulating crop stress responses. Apart from ROS, reactive nitrogen species (RNS), reactive sulfur species (RSS), and reactive carbonyl species (RCS) also play critical roles in signal transduction and participate in plant responses to abiotic stresses [102]. Therefore, ROS remains a focal point in the study of plant stress biology.

### 5.2. The Reason of ROS Accumulation

ROS accumulation occurs in plant cells caused by various environmental stresses, such as drought, high light intensity, extreme temperatures, pollutants, and pathogen attacks. These stresses disrupt cellular homeostasis, leading to an imbalance between ROS production and antioxidant defense mechanisms, resulting in ROS accumulation.

Under drought stress, an excessive accumulation of ROS can lead to oxidative stress reactions, causing lipid peroxidation, cell damage, metabolic disruption, and reduced plant productivity [103]. Firstly, drought induces stomatal closure, reducing CO_2_ entry into cells, lowering photosynthetic rates, hindering carbon fixation, and thereby causing a continuous reduction in molecular oxygen, resulting in excessive ROS production [93]. Secondly, protein and membrane system denaturation caused by photorespiration, enzyme inactivation in the tricarboxylic acid cycle (TCA cycle), and reduced carboxylation efficiency during drought is also associated with excessive ROS accumulation [104].

### 5.3. Detoxification of ROS

**Figure 3:**
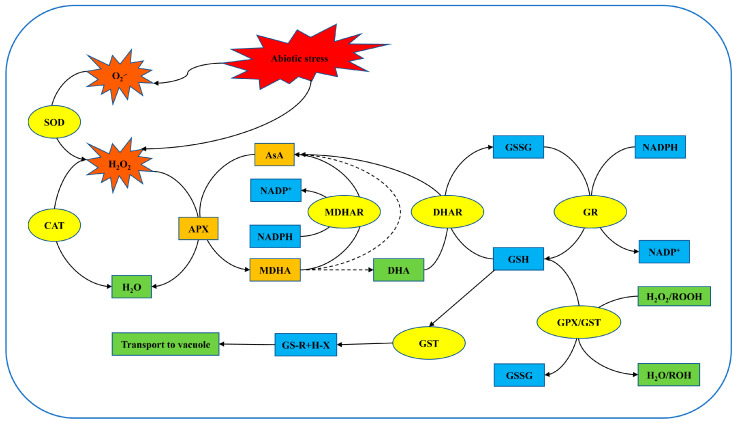
The mechanism of the antioxidant system to eliminate ROS. The solid line is the effect of antioxidant enzymes, and the dotted line is the effect of non-enzyme substances. SOD: Superoxide dismutase; CAT: Catalase; APX: Ascorbate peroxidase; AsA: Ascorbate; MDHA: Monodehydroascorbate; MDHAR: Monodehydroascorbate reductase; DHAR: Dehydroascorbate reductase; DHA: Dehydroascorbate; GSH: Glutathione; GST: Glutathione S-transferase; GSSG: Oxidized glutathione; GR: Glutathione reductase; GPX: Glutathione peroxidase.

To withstand adverse environments, plants must regulate the homeostasis of ROS within their bodies to prevent toxicity caused by excessive ROS accumulation. ROS detoxification is maintained by an antioxidant defense system composed of both enzymatic and non-enzymatic substances. Antioxidants directly or indirectly eliminate ROS or control their production [105]. Enzymes involved in ROS detoxification include superoxide dismutase (SOD), catalase (CAT), glutathione S-transferase (GST), peroxidases (POD), and glutathione peroxidase (GPX), as well as four enzymes in the Ascorbate-Glutathione (AsA-GSH) cycle: ascorbate peroxidase (APX), glutathione reductase (GR), monodehydroascorbate reductase (MDHAR), and dehydroascorbate reductase (DHAR). In addition to enzymes, non-enzymatic substances also participate in ROS scavenging, such as ascorbate (AsA), carotenoids, α-tocopherol (vitamin E), glutathione (GSH), phenolic acids, and flavonoids, among other low-molecular-weight compounds [106]. Non-enzymatic substances and antioxidant enzymes synergistically regulate the redox state of cells, suppressing excessive ROS production (Figure 3).

In plants, SOD is directly associated with stress responses. It initiates the first line of defense by dismutating O_2_^•−^ into H_2_O_2_ [107]. The generated H_2_O_2_ can be further converted into H_2_O by enzymes, such as CAT, APX, and GPX, or catalyzed in the AsA-GSH cycle [108]. CAT is a tetrameric heme-containing enzyme involved in detoxifying ROS, capable of converting 26 million molecules of H_2_O_2_ to H_2_O in a minute [93]. POD primarily oxidizes phenols (PhOH) to produce phenoxyl radicals (PhO·) and reduces H_2_O_2_ to H_2_O upon accepting electrons. In the absence of AsA, PhO· reactions lead to suberin, lignin, and quinones, whereas in the presence of AsA, PhO· reacts with AsA to form monodehydroascorbate (MDHA), which subsequently forms dehydroascorbate (DHA) [109].

In plants, APX is capable of scavenging excess H_2_O_2_ generated by oxidative stress and producing MDHA [110]. The generated MDHA is reduced back to AsA via NADPH-dependent Flavin Adenine Dinucleotide (FAD)-dependent monodehydroascorbate deductase (MDHAR). MDHA can also be further reduced to DHA by non-enzymatic substances, and DHA is then restored to AsA by GSH-dependent dehydroascorbate reductase (DHAR) [111]. During this process, GSH is oxidized to oxidized glutathione (GSSG), which is subsequently reduced back to GSH by NADPH-dependent glutathione reductase (GR) [112].

In plant cells, the AsA-GSH cycle, also known as the Asada–Halliwell pathway, is the primary antioxidant defense pathway for scavenging H_2_O_2_. Among non-enzymatic antioxidants, AsA and GSH are the most abundant soluble antioxidants in higher plants. Many pathways involved in phytohormone biosynthesis are regulated by AsA [113], and they play crucial roles as electron donors in directly scavenging ROS through the AsA-GSH cycle [114].

α-Tocopherol protects chloroplasts and maintains photosynthesis by scavenging ROS (primarily ^1^O_2_ and ^•^OH) [115]. Carotenoids react with ^•^OH, O_2_^•−^, and ROO^•^ radicals, thereby protecting the photosystem and stabilizing thylakoid membranes to reduce cellular ROS levels [116]. Flavonoids have significant potential in scavenging free radicals and alleviating lipid peroxidation-induced cellular damage. They are upregulated under abiotic stress, activating antioxidant defense mechanisms to mitigate oxidative stress [117]. Hydroxycinnamic acids primarily act as chelators and scavengers of ^•^OH, O_2_^•−^, and ROO^•^ radicals, exerting their antioxidant activity [105].

The response mechanisms of antioxidant defense adapting to drought stress have been extensively studied. Research indicated that the overexpression of *ZmWRKY79* in Arabidopsis reduced stomatal aperture, slowed down water loss, and enhanced survival rates under drought stress [81]. ZmWRKY79 also promoted ROS scavenging, reduced accumulation of H_2_O_2_ and MDA, and enhanced antioxidant enzyme activity [81]. The overexpression of *ZmWRKY106* in transgenic Arabidopsis improved tolerance to drought and high-temperature stress, increasing activities of SOD, POD, and CAT under drought stress, and reducing ROS levels in the transgenic lines [118]. Studies on drought tolerance in two sorghum varieties revealed significant increases in SOD and APX activities under drought stress, mitigating oxidative damage and enhancing drought resistance [119]. These results highlight the indispensable role of the antioxidant system in plants combating drought stress.

## 6. The Regulatory Role of Phytohormone in Drought Stress

Plants have evolved physiological and biochemical mechanisms to cope with drought stress, maintaining stable cell water potential and relative water content [120]. Phytohormone act as regulatory factors throughout the life cycle in plants, playing crucial roles [121]. Phytohormones, like abscisic acid (ABA), jasmonic acid (JA), ethylene (ET), and salicylic acid (SA), participate in osmotic regulation processes related to drought [122]. Upon sensing stress signals, these hormones activate various physiological and developmental processes, such as stomatal closure, root growth, and the accumulation of osmolytes, to modulate drought stress [123].

### 6.1. ABA

ABA is an important regulatory factor in plant development, influencing traits, such as embryo maturation, seed germination, and dormancy [124]. Additionally, ABA serves as a major stress-responsive hormone that mitigates the adverse effects of drought stress on plants [125]. Research indicated that osmotic stress enhanced ABA synthesis, thereby activating gene expression related to adaptive physiological changes [126].

The core of the ABA signaling pathway consists of ABA receptors PYR/PYL/RCARs, ABA co-receptor type 2C protein phosphatases (PP2Cs), and SnRK2s [127]. ABA-dependent signaling pathways have multiple branches regulated by various transcription factors such as MYB, MYC, and NAC, as well as calcium-dependent protein kinases (CDPKs) [128].

Stomata serve as the gateway for gas and water exchange between plants and the atmosphere, and ABA participates in regulating their opening and closing. Stomatal closure is a critical physiological process to reduce water loss under drought conditions, where ABA triggers the closure by promoting the efflux of K^+^ from guard cells and clearing osmotic regulators [129]. Ion channels on the plasma membrane and vacuolar membrane, including channels for inward and outward transport of K^+^ and Ca^2+^, are involved. ABA promotes the opening of ion channels on the vacuolar and plasma membranes, releasing ions into the cell, leading to H^+^-ATPase inactivation. As a result, guard cells shrink due to water loss, causing stomatal closure, thereby preventing further loss of cellular water [130].

### 6.2. JA

Jasmonic acid (JA) is a phytohormone derived from α-linolenic acid. JA and its active derivative MeJA play crucial roles in regulating plant responses to abiotic stress.

In the JA signaling pathway, Jasmonate ZIM domain proteins (JAZ) act as switches for JA signaling. Under normal conditions when JA is absent, JAI3/JAZ proteins bind to various transcription factors and inhibit their activity. Under stress conditions, when JA is present, JAZ proteins are degraded, leading to the activation of these transcription factors that upregulate the expression of stress-responsive genes [131]. JA mediates plant drought tolerance through pathways such as stomatal closure and ROS scavenging. For instance, treatment of Arabidopsis with the JA precursor 12-oxo-phytodienoic acid (12-OPDA) resulted in stomatal closure [132]. Moreover, elevated levels of 12-OPDA led to reduced stomatal density, enhancing Arabidopsis drought tolerance [132].

Research found that exogenous application of JA could participate in the response to drought stress, enhancing antioxidant activity under drought conditions. For example, under drought stress, exogenous JA significantly increased the activities of GR, APX, DHAR, and MDHAR in wheat seedlings [133]. The transient accumulation of JA in roots led to an increase in ABA levels, which together played roles in stress responses [134]. Generally, phytohormones do not act in isolation but rather interact synergistically or antagonistically to regulate plant growth, development, and environmental adaptation. Similar to ABA signaling, JA serves as a central hub for initiating various physiological and biochemical processes in response to drought stress.

### 6.3. SA

Salicylic acid (SA) is a phenolic compound that plays roles in plant growth, development, and response to biotic stresses [135]. The isochorismate synthase (ICS) pathway is the major biosynthetic route for SA, where isochorismate is converted to SA by ICS. The overexpression of *ICS1* increased SA accumulation, aiding plants in combating environmental stresses [136].

SA accumulation leads to the monomerization of the non-expressor of pathogenesis-related genes 1 (NPR1) oligomer. Upon entry into the nucleus, monomeric NPR1 binds SA, undergoes conformational changes, and releases its C-terminal activation domain, thereby triggering the transcription of target genes [137]. SA-activated genes, including WRKYs, enhance the ability of plants to resist abiotic stresses.

Under drought stress, the SA content significantly increases in plants. For instance, the SA content in barley roots doubled during drought conditions [138]. Arabidopsis mutant *myb96-1d* accumulated SA endogenously and exhibited strong drought tolerance [139]. Similarly, under drought stress, SA accumulation leads to stomatal closure, thereby enhancing drought tolerance in plants such as maize and Arabidopsis [140].

### 6.4. Ethylene

Ethylene (ET) is a naturally occurring gaseous hormone that plays a positive role in regulating plant drought tolerance. For example, the expression of soybean ethylene response factor *GmERF3* was induced by drought, ET, ABA, SA, and JA [141]. Tobacco plants overexpressing *GmERF3* showed higher proline and SS contents compared to the wild type, exhibiting enhanced drought tolerance [141]. *ZmARGOS8* acted as a negative regulator in the ET response; the overexpression of *ZmARGOS8* reduced plant sensitivity to ethylene and enhanced drought tolerance [142]. Experiments have also demonstrated that ethylene release regulated the expression of wax synthesis regulatory genes *ZmERE* and wax synthesis genes *ZmGL1*, *ZmGL15*, *ZmFDH1*, and *ZmFAE1*, promoting the accumulation of epicuticular wax in maize seedlings under drought stress, resulting in reduced leaf water loss, decreased concentrations of MDA and H_2_O_2_, increased proline accumulation, and enhanced SOD, POD, and CAT activities [143]. These results indicate that ET can maintain the necessary water status and membrane stability for plant growth, thereby improving maize tolerance to drought stress.

Each phytohormone associated with drought stress plays a crucial role in enhancing drought tolerance. Moreover, these phytohormones do not act in isolation but rather regulate each other’s biosynthesis and responses through synergistic or antagonistic interactions [144]. A study indicates that ABA shares common functional components with other phytohormones, thereby participating in crosstalk to maintain cellular homeostasis [145]. For instance, ABA, JA, SA, BR, and ET all participate in stomatal closure [146]. Methyl Jasmonate (MeJA), similar to ABA, induces the production of ROS and NO and activates S-type anion channels to regulate stomatal closure. In ABA-insensitive mutants such as *abi1-1* and *abi2-1*, MeJA does not induce stomatal closure but stimulates ROS and NO production [147]. Therefore, phytohormones do not act independently but interact with each other to collectively regulate plant responses to drought stress.

### 6.5. Cytokinins

Cytokinins (CKs) are a class of phytohormones that regulate cell division and differentiation. They play a crucial role not only in plant growth and development but also in the response to abiotic stress [148,149,150]. CKs are considered one of the primary regulatory factors, and mutants with alterations in cytokinin synthesis and signaling pathways exhibit dwarf phenotypes [151]. Abiotic stresses such as drought and salt stress affect the content, transport, and signaling activity of CKs in plants, and CKs negatively regulate the response to these stresses [149,152,153,154]. The reduction in cytokinin levels under stress conditions may be due to the suppression of the expression of cytokinin synthesis genes *IPT1*, *IPT3*, and *IPT5* and the promotion of the expression of cytokinin oxidase/dehydrogenase genes *CKX1*, *CKX3*, and *CKX6* [153]. Osmotic stresses such as drought induce the expression of the *AHK1* gene, while low temperature, drought, and salt stress can induce the expression of *AHK3* [152,155]. Additionally, drought and salt stress can induce the expression of *ARR5*, *ARR15*, and *ARR22* [152].

## 7. sRNA Involved in Drought Stress

Small RNA (sRNA) is a class of short non-coding RNAs that are widely present in eukaryotes. They play a crucial role as important regulatory elements in gene expression, influencing processes such as growth and development, environmental responses, and metabolism [156,157].

Numerous studies have shown that sRNAs are involved in the stress response of plants. Previous research analyzing whole-genome sRNA sequencing data from different species under drought and control conditions found that the expression levels of many miRNAs and tas-siRNAs changed significantly, indicating that sRNAs could respond to drought stress [158,159,160]. Additionally, the functional analysis of conserved miRNA target genes revealed that many of these target genes could influence the response to drought in plants. Therefore, miRNAs can further regulate the expression of target genes by altering their own expression levels in response to drought.

miR169 can respond to drought stress by regulating the NF-YA gene family members. The overexpression of miR169c in Arabidopsis was shown to enhance drought resistance [161]. In rice, knocking out miR166 could affect xylem development and cause leaf curling, enhancing drought tolerance in the plants [162]. In alfalfa, miR156 could improve drought resistance by silencing *SPL13* [163].

In addition to the conserved miRNA families, the functions of certain species-specific miRNAs in drought response have also been gradually revealed. For instance, miRNA1916 in tomato and potato acted as a negative regulator of drought response by mediating the cleavage of mRNAs for histone deacetylases (HDAC) and strictosidine synthase (STR) genes [164]. In legumes, miR1514a responded to drought by influencing the formation of phasiRNAs through the regulation of NAC transcription factor expression levels [165].

In addition to miRNAs, analysis of whole-genome sRNA sequencing has also revealed that a large number of heterochromatic siRNAs exhibit significant changes in expression following drought treatment [158]. Additionally, the methylation levels in many RdDM target regions of the genome also undergo significant changes under drought stress, suggesting that these heterochromatic siRNAs may play an important role in the transcriptional regulation of the drought response [158].

Studies have shown that changes in methylation levels in certain regions caused by RdDM during abiotic stress responses can be partially transmitted to the next generation, affecting the memory response to stress. However, analysis of DNA methylation levels in successive generations of Arabidopsis after drought stress has indicated that the drought memory response in offspring is not related to DNA methylation, and the transmission of DNA methylation is not influenced by drought [166,167].

## 8. Conclusions and Prospects

Drought resistance is a complex and multi-gene-controlled trait. With the development of molecular biology and genomics technologies, research on drought-resistant regulation pathways has become increasingly in depth, providing new strategies and methods for plant breeding. i. The overexpression or knockout of a single gene may affect a large number of downstream genes and may result in different responses to stress and growth and development. For example, the overexpression of *DREB2* endowed transgenic plants with higher drought resistance, but it was also accompanied by a decrease in the yield [168]. Therefore, in future research, tissue-specific promoters and stress-induced promoters can be used to drive different genes in plants. ii. Due to the absence of exogenous genes in the varieties obtained through gene-editing breeding, the advantages of gene-editing technology for crop improvement are obvious. It is necessary to accelerate the research of gene-editing technology in promoting drought-resistant plant breeding. iii. With the continuous development of bioinformatics, bioinformatics tools and big data analysis can be used to predict and validate new regulatory factors at the whole-genome level. Exploring important regulatory factors that can increase or not affect growth and development phenotypes such as yield and improve plant stress resistance. Studying their potential roles in drought resistance regulation can provide a theoretical basis for cultivating drought-resistant varieties. In summary, through in-depth research on the functions of drought-resistant factors, and combined with modern biotechnology, it is expected that new crop varieties will be cultivated that are more drought-resistant and high-yielding, contributing to ensuring food security and sustainable agricultural development.
